# ZP3 is Required for Germinal Vesicle Breakdown in Mouse Oocyte Meiosis

**DOI:** 10.1038/srep41272

**Published:** 2017-02-01

**Authors:** Lei-Lei Gao, Chun-Xiang Zhou, Xiao-Lan Zhang, Peng Liu, Zhen Jin, Gang-Yi Zhu, Yang Ma, Jing Li, Zhi-Xia Yang, Dong Zhang

**Affiliations:** 1 State Key Laboratory of Reproductive Medicine, Nanjing Medical University, Nanjing 211166, China.

## Abstract

ZP3 is a principal component of the zona pellucida (ZP) of mammalian oocytes and is essential for normal fertility, and knockout of ZP3 causes complete infertility. ZP3 promotes fertilization by recognizing sperm binding and activating the acrosome reaction; however, additional cellular roles for ZP3 in mammalian oocytes have not been yet reported. In the current study, we found that ZP3 was strongly expressed in the nucleus during prophase and gradually translocated to the ZP. Knockdown of ZP3 by a specific siRNA dramatically inhibited germinal vesicle breakdown (GVBD) (marking the beginning of meiosis), significantly reducing the percentage of MII oocytes. To investigate the ZP3-mediated mechanisms governing GVBD, we identified potential ZP3-interacting proteins by immunoprecipitation and mass spectrometry. We identified Protein tyrosine phosphatase, receptor type K (Ptprk), Aryl hydrocarbon receptor-interacting protein-like 1 (Aipl1), and Diaphanous related formin 2 (Diaph2) as potential candidates, and established a working model to explain how ZP3 affects GVBD. Finally, we provided preliminary evidence that ZP3 regulates Akt phosphorylation, lamin binding to the nuclear membrane *via* Aipl1, and organization of the actin cytoskeleton *via* Diaph2. These findings contribute to our understanding of a novel role played by ZP3 in GVBD.

Germinal vesicle breakdown (GVBD) is the first prerequisite process for successful meiosis, and is determined by at least two consecutive component steps. First, for GVBD to occur, all essential preceding events need to be performed successfully. Failure in any process could activate the G2/M checkpoint and arrest the oocytes at the prophase (GV) stage. Second, at the onset of GVBD, the major configuration of the GV has to be properly reorganized^1^,^2^,^3^.

The first component is, in essence, a matter of nuclear maturation; the governing regulatory mechanism is very complicated, but two major events, chromatin configuration and nuclear envelope assembly, are well studied^1^,^2^,^3^. A fully competent GV oocyte needs to be transcriptionally quiescent, such that the chromatin can be converted from non-surrounded nucleus (NSN) to surrounded nucleus (SN) chromatin, which is closely related to the levels of cAMP and is regulated by several important kinases^1^,^2^,^3^,^4^,^5^. Lamins (A, B and C) also play important roles in nuclear envelope assembly^6^,^7^. Mature lamin A is formed from prelamin A after a series of post-translational modifications. Farnesylation is the addition of a nonpolar farnesyl residue, promoting membrane binding, and lamin A farnesylation is a critical step in its correct localization to the nuclear membrane^8^,^9^. In *Xenopus* egg extracts, importin alpha and beta promote lamin assembly by modulating the interaction of lamins with lamin-binding proteins^10^,^11^,^12^. However, this has not yet been investigated in mammalian oocytes. Lamins play an important role in many nuclear events, including DNA replication, RNA transcription, nuclear and chromatin organization, cell cycle regulation, cell development and differentiation, nuclear migration, and apoptosis, as well as nuclear envelope assembly^6^,^7^,^8^,^9^.

Regarding the second step, it is well known that GVBD requires the cooperative action of diverse signaling molecules. In ovaries, high levels of cAMP arrest oocytes in the GV stage of meiosis I. Progesterone, in some species, then releases oocytes from this arrest by activation of maturation promoting factor (MPF) by the dual specific phosphatase cell division cycle 25 (Cdc25). The regulatory component of MPF is cyclin B and the catalytic component is cyclin-dependent kinase 1 (Cdk1), and upon activation, Thr14 and Tyr15 of Cdk1 are dephosphorylated by Cdc25^1^,^2^,^3^,^4^,^5^. *In-vitro* maturation (IVM) of mouse oocytes and GVBD are, however, not dependent on progesterone. In addition, the nuclear envelope has to be disassembled in response to specific signaling events during GVBD in mouse oocytes, and the disassembly of lamin A/C is regulated through phosphorylation at Ser22 and Ser392 residues by Cdk1^13^,^14^,^15^,^16^. Normal cytoskeletal dynamics are also essential for GVBD. Treatment of cycloheximide (CHX)- arrested bovine oocytes with the actin-stabilizing agent jasplakinolide significantly delayed GVBD after CHX release^17^. The apparent reason for this was that proper actin dynamics are important for Cyclin B1 translation, probably by making Cyclin B1 mRNA more accessible^17^. Therefore, decreased actin dynamics can reduce MPF activity, as has been observed for *Xenopus* oocytes^18^. Kinesin family member 5B (Kif5B) is a microtubule and chromosome arm-associated kinesin and is important for maintaining chromosomal stability in mitotic cells. Kif5B knockdown by siRNA induced significant delay in GVBD, indicating that the microtubule cytoskeleton outside the nucleus is important for chromatin reorganization^19^. Early spindle microtubules impinging on the nuclear envelope are also involved in breakdown of the nuclear envelope by inducing lamin B1 disassembly^20^.

ZP3 is one of the major components of the zona pellucida, which surrounds the oocyte membrane (oolemma) to prevent polyspermy and the entry of heterogenous sperm. Specifically, ZP3 plays key roles in the binding of sperm to the surface of the zona pellucida and the subsequent acrosome reaction^21^,^22^,^23^,^24^,^25^. Although ZP3 has two ZP domains similar to those of ZP1 and ZP3, other regions of ZP3 are distinct^26^,^27^; and newly-synthesized cytoplasmic ZP3 is transported to the zona pellucida independently of ZP2^28^,^29^. However, no reports have ascribed new functions to ZP3 in addition to its classical role as a component within the zona pellucida.

In the present study, we found that ZP3 was concentrated within the nucleus at the GV stage and then dramatically decreased around the chromosomal region after GVBD. This dynamic change in ZP3 localization urged us to investigate whether ZP3 was also important in normal GVBD.

## Results

### ZP3 is concentrated in the nucleus before the resumption of meiosis and then relocates to the zona pellucida during meiosis

**Figure 1:**
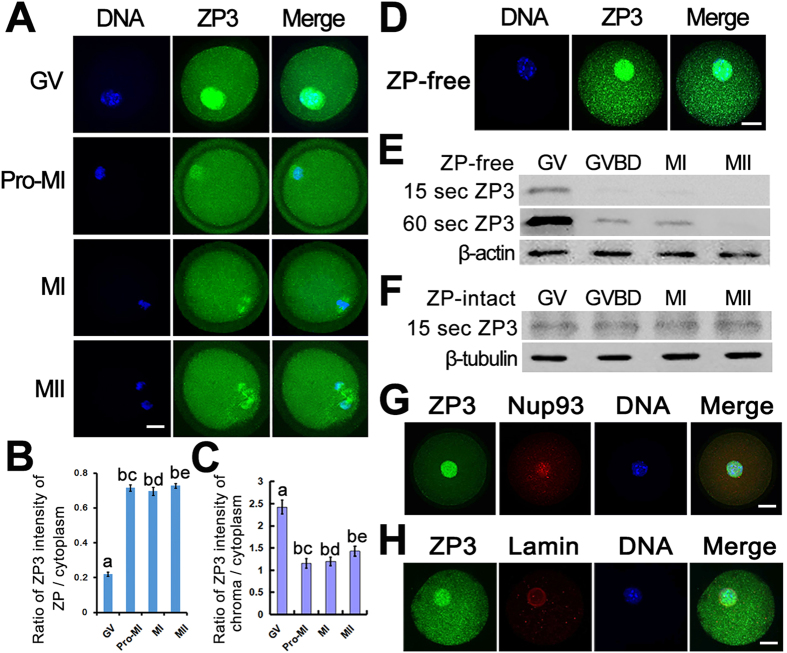
ZP3 is concentrated in the nucleus before meiosis then relocates to the zona pellucida during meiosis. (**A**) ZP3 Immunofluorescene in intact mouse oocytes showed that Gm364 concentrates within nucleus region before GVBD and levels up within zona pellucida (ZP) after GVBD in mouse oocytes. (**B**) Ratio of ZP3 fluorescence intensity of ZP/cytoplasm at different meiotic stages showed that ZP3 abundance within ZP was very low at germinal vesicle (GV) stage and significantly leveled up after GVBD. (**C**) Ratio of ZP3 fluorescence intensity of chroma/cytoplasm at different meiotic stages showed that ZP3 abundance within chromosome region was very high at germinal vesicle (GV) stage and significantly decreased after GVBD. “Chroma” here is a broad definition of chromatin region (GV stage) or regions of chomosome with surrounding spindle microtubules (GVBD, MI, MII). (**D**) In ZP-free GV oocytes, ZP3 still highly concentrated within nucleus region. (**E**,**F**) Western blot showed that total ZP3 in ZP-intact oocytes remained constant, while in ZP-free oocytes, ZP3 amount within cytoplasm was the highest at GV stages, then dramatically decreased after GVBD. In (**E)**, two images of the same ZP3 blot at different exposure time, 15 sec and 60 sec were shown. (**G**,**H**) At GV stage, ZP3 localization within nucleus was distinct from Nup93 or lamin A/C. DNA in blue, ZP3 in green, Nup93 or Lamin A/C in red. Different letters in (**B**,**C**) indicate significant difference. Scale bar, 20 μm.

We observed unexpected ZP3 staining in GV oocytes, with signal strongest in the nucleus and weakest in the zona pellucida (ZP). We largely repeated this staining pattern with another commercial ZP3-specific antibody (Fig. S1). We systematically checked its localization at each stage during meiosis and found that the intensity of the chroma region (here “chroma” refers to a broad definition of the chromosome and surrounding spindle microtubules) dramatically decreased at GVBD and remained low during MI and MII stages (Fig. 1A and B). The remaining cytoplasmic ZP3 co-localized with spindle microtubules and nocodazole-treatment showed that the ZP3 localization within spindles was microtubule dependent (Fig. 1A and C; Fig. S2). In contrast, the staining in the zona pellucida significantly increased during meiosis (Fig. 1A and C). To further examine the cytoplasmic localization of ZP3 we performed immunostaining experiments in ZP-free oocytes and found that ZP3 was still highly concentrated within the nucleus at the GV stage (Fig. 1D). We also performed western blot analyses of ZP3 expression in ZP-free oocyte lysates and obtained the same results; *i.e.,* ZP3 protein levels were high at the GV stage and dramatically declined during GVBD and MI, and almost completely disappeared by the MII stage (Fig. 1E). In contrast, ZP3 expression remained constant in intact oocytes at different stages (Fig. 1F). We also performed co-immunostaining of ZP3 with other nuclear proteins, including Nucleoporin 93 (Nup93) and lamin A/C, and found that ZP3 only partially overlapped with Nups and lamins (Fig. 1G and H).

### ZP3 is important for germinal vesicle breakdown, spindle organization, and oocyte maturation

**Figure 2:**
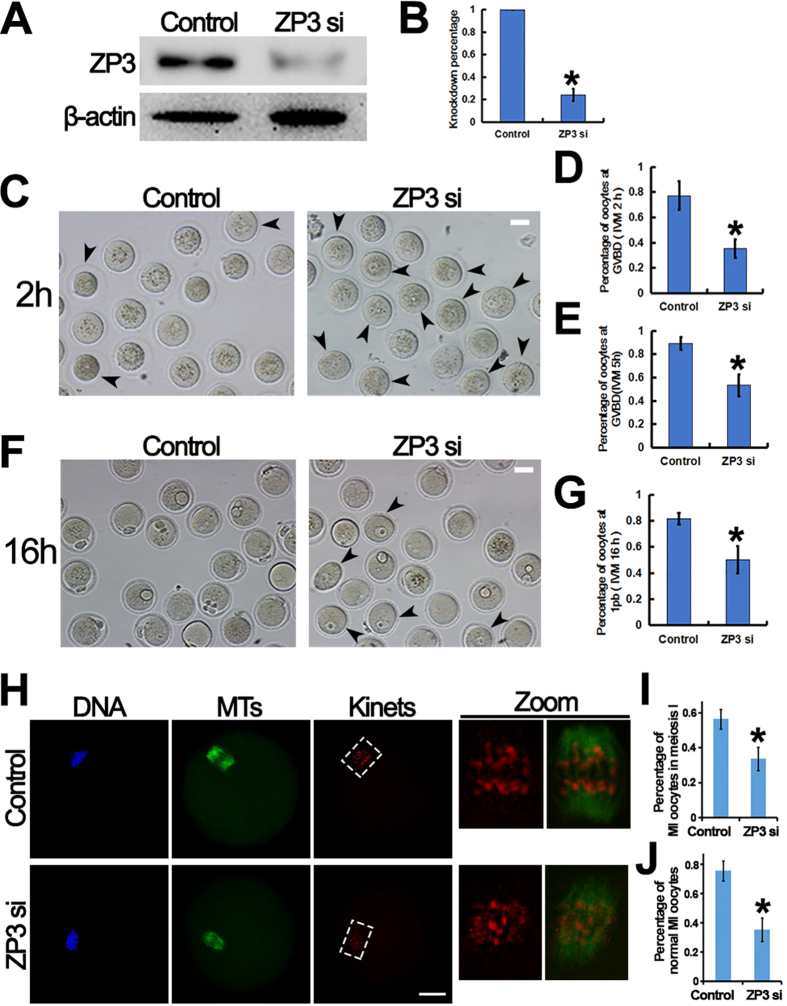
ZP3 is important for germinal vesicle breakdown, spindle organization, and oocyte maturation. (**A**,**B**) ZP3 was able to be efficiently knocked down by specific siRNA. (**C**,**D**) At 2 h of IVM after ZP3 knockdown, percentage of GVBD significantly decreased. GV oocytes are arrow pointed. (**E**) At 5 h of IVM after ZP3 knockdown, percentage of GVBD still significantly decreased. (**F**,**G**) At 16 h of IVM after ZP3 knockdown, percentage of 1pb oocytes was significantly lower than control. GV oocytes are arrow pointed. (**H**–**J**) At 8 h of IVM after ZP3 knockdown, although fluorescence intensity of spindle microtubules didn’t change, kinetochore alignment and kinetochore-microtubule attachment was severely disturbed. Kinetochore regions (white dot-line square) of single kinetochore channel or double microtubule & kinetochore channel were zoomed in to show the microtubule-kinetochore attachment. DNA in blue, MTs in green, kinetochores in red. Significant difference are asterik (*) labeled. Scale bar, 20 μm.

Based on our findings, we hypothesized that ZP3 was involved in GVBD. To investigate this, we knocked down ZP3 in GV oocytes using specific siRNAs (Fig. 2A and B). As expected, ZP3 knockdown significantly decreased the percentage of oocytes undergoing GVBD (controls, 77.32%; ZP3 knockdown, 35.42%) (Fig. 2C and D) at 2 h of IVM; and at 5 h after knockdown, nearly 50% of oocytes were still in the GV stage in the ZP3-knockdown group (percentage of GVBD: controls, 89.35%; ZP3 knockdown, 53.27%) (Fig. 2E). At 16 h, the percentage of MII oocytes in the ZP3- knockdown group was significantly lower than controls (controls, 81.61%; ZP3 knockdown, 50.12%) (Fig. 2F and G).

Since ZP3 is involved in meiotic entry, we also checked whether spindle organization was affected by ZP3 knockdown. At 8 h of IVM, although ZP3 knockdown did not affect microtubule intensity within the spindles, there were significantly lower percentage of MI oocytes (percentage of MI oocytes, controls *vs*. ZP3 knockdown, 56.48% *vs*. 33.71%) (Fig. 2H and I); and significantly more MI oocytes showed aberrant kinetochore-microtubule attachments as shown by the randomly distributed kinetochores and kinetochore-MT fibers within chromosomal regions (percentage of normal MII oocytes, controls *vs*. ZP3 knockdown, 75.38% vs. 35.19%) (Fig. 2H and J).

### ZP3 appears to function in germinal vesicle breakdown in multiple ways

**Figure 3:**
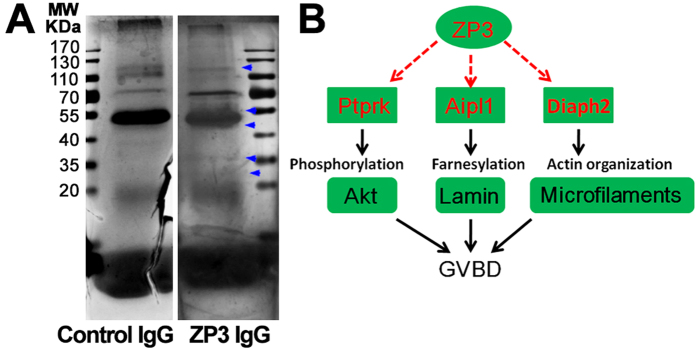
ZP3 might function in germinal vesicle breakdown in multiple ways. (**A**,**B**) To identify ZP3-interacting proteins, Immunoprecipitation was done with control IgG or ZP3 antibody in lysate of ZP-free GV oocytes and then samples were separated on a SDS-PAGE gel and subsequently subject to silver staining. Presumable bands distinct from control (blue arrow head pointed) were submitted for MALDI-TOF-MS. Besides ZP3 itself, Ptprk (protein tyrosine phosphatase, receptor type K), Aipl1 (aryl hydrocarbon receptor-interacting protein-like 1) and Diaph2 (diaphanous related formin 2) were identified as major ZP3-interacting proteins. An working model was established based on reference search & software prediction: ZP3 might regulate Ptprk, which could regulate Akt activity by phosphorylation; ZP3 might regulate Aip1, which could regulate lamin farnesylation; ZP3 might regulate Diaph2, which could regulate actin cytoskeleton organization.

To further investigate how ZP3 promoted GVBD, we used a ZP3 antibody to perform immunoprecipitation experiments in ZP-free GV oocyte lysates. Immunoprecipitated proteins were separated by SDS-PAGE and silver staining was used to visualize and select distinct bands of interest for protein identification by mass spectrometry (Fig. 3A, arrows). We identified ZP3 at the expected size, as well as other proteins with known functions. By consulting the literature, we established a working model where ZP3 interacted with protein tyrosine phosphatase receptor-type K (ptprk) to regulate Akt phosphorylation; with aryl hydrocarbon receptor-interacting protein-like 1 (Aipl1) to regulate lamin farnesylation; and with diaphanous-related formin 2 (Diaph2) to regulate actin cytoskeleton remodeling. Collectively, these interactions promoted GVBD (Fig. 3B).

### ZP3 affects GVBD by regulating Akt phosphorylation, Aipl1 and Diaph2 distribution

**Figure 4:**
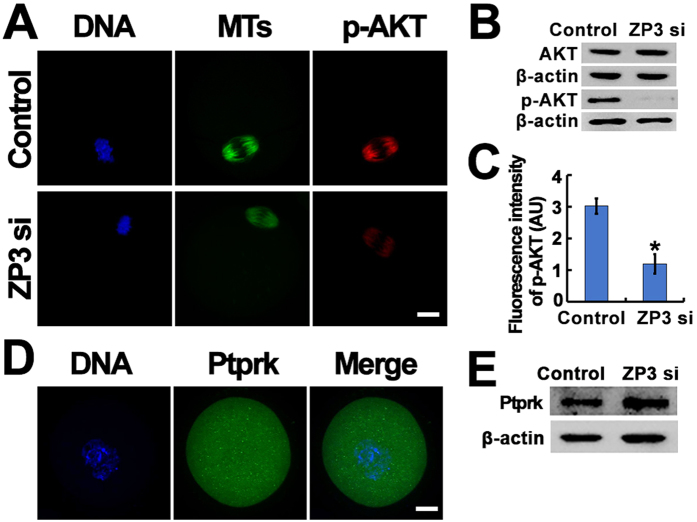
ZP3 might affect GVBD by regulating Akt phosphorylation. (**A**–**C**) ZP3 knockdown significantly decreased phosphorylated Akt (p-Akt, s473). DNA in blue, MTs in green, p-AKT in red. (**D**,**E**) ZP3 knockdown didn’t alter Ptprk level. DNA in blue, Ptprk in green. Significant difference are asterik (*) labeled. Scale bar, 20 μm.

To validate our model, we performed further experiments. First, both western blot and immunostaining experiments showed that p-Akt (S473) significantly decreased after ZP3 knockdown (Fig. 4A–C), while total Akt levels did not change. However, Ptprk, the known upstream kinase of Akt, did not show a particularly distinguishable staining pattern, nor did the total protein level change (Fig. 4D and E). Unfortunately, we were not able to locate a commercial antibody against the modified form of Ptprk, so we could not determine whether or how it had changed.

**Figure 5:**
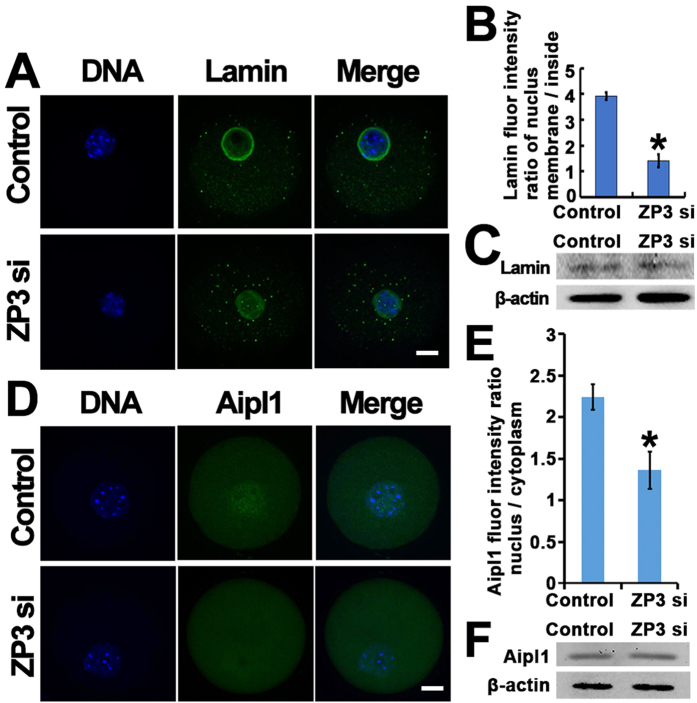
ZP3 might affect GVBD by regulating lamin and Aipl1 distribution. (**A**–**C**) ZP3 knockdown significantly decreased lamin A/C intensity at nuclear membrane while increased lamin A/C intensity within nuclear cytoplasm. DNA in blue, lamin A/C in green. (**D**–**F**) ZP3 knockdown significantly decreased Aipl1 concentration within nucleus without affecting the total Aipl1 level. DNA in blue, Ptprk in green. Significant difference are asterik (*) labeled. Scale bar, 20 μm.

Second, although total lamin levels did not change, intensity of lamin A/C staining at the nuclear membrane significantly decreased while intensity within the nucleus significantly increased in the ZP3-knockdown group. The ratio of lamin A/C intensity at the nuclear membrane *vs*. nuclear cytoplasm reflected this more clearly (Fig. 5A–C). We then further assessed whether ZP3 affected Aipl1 as proposed in our model (Fig. 3B). Interestingly, Aipl1 was enriched within the nucleus in a manner similar to that for ZP3; and ZP3 knockdown greatly diminished the concentration of Aipl1 within the nucleus, while total Aipl1 levels did not change (Fig. 5D–F).

**Figure 6:**
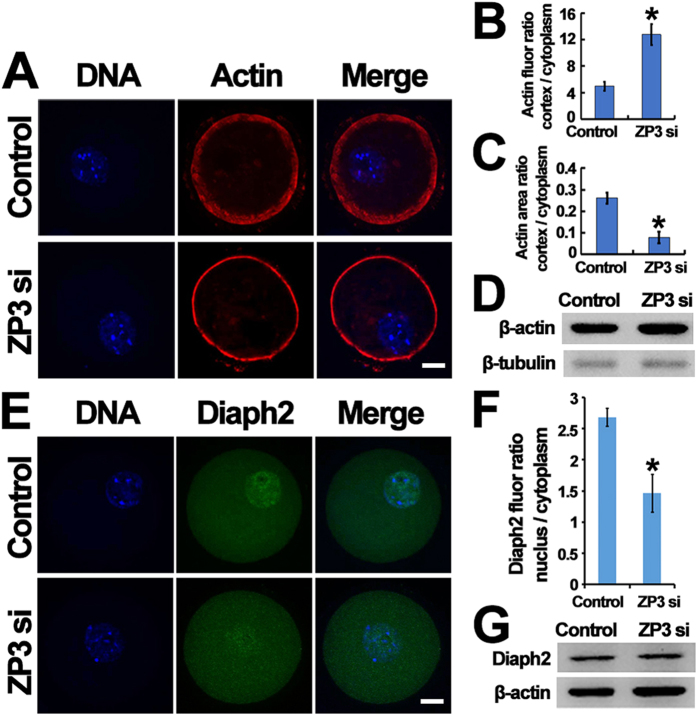
ZP3 might affect GVBD by regulating Diaph2 distribution. (**A**–**D**) ZP3 knockdown significantly decreased cortical microfilament (F-actin) area and increased microfilament intensity while total actin level remained the same. DNA in blue, F-actin in red. (**E**–**G**) ZP3 knockdown significantly decreased Diaph2 concentration within nucleus without affecting the total Diaph2 level. DNA in blue, Diaph2 in green. Significant difference are asterik (*) labeled. Scale bar, 20 μm.

Third, although the total actin level did not change, the organization of actin filaments (F-actin) was altered greatly by ZP3 knockdown (Fig. 6A and D). In control oocytes, F-actin was distributed more widely and actin fluorescence was significantly lower. However, in ZP3-knockdown oocytes, F-actin exhibited a narrower distribution and F-actin fluorescence intensity was increased (Fig. 6A). The area encompassed by the F-actin distribution and its staining intensity were quantified as ratios of cortical to cytoplasmic areas (clearly reflecting changes [Fig. 6B and C]), while actin levels remained the same (Fig. 6D). Next, we further evaluated whether ZP3 also affected Diaph2 as proposed in our model (Fig. 3B). Interestingly, Diaph2 also manifested a similar nuclear localization, and the concentration of Diaph2 within the nucleus dramatically decreased, although the total Diaph2 levels remained unchanged (Fig. 6E–G).

## Methods

### Chemicals, reagents, and animals

Chemicals and reagents were obtained from Sigma unless otherwise stated. ICR mice were obtained from Vitalriver experimental animal technical co., LTD, Beijing. All animal experiments were approved by the Animal Care and Use Committee of Nanjing Medical University and were performed in accordance with institutional guidelines.

### Antibodies

Mouse monoclonal anti-β-actin (Cat#: A5316–100) was purchased from Santa Cruz (St. Louis, MO, USA). Rabbit polyclonal ZP3 (H-300) (Cat#: sc-25802) was purchased from Santa Cruz (Dallas, Texas, USA). Mouse monoclonal anti-β-tubulin (Cat#: sc-5274) was purchased from Santa Cruz (Dallas, Texas, USA). Goat polyclonal lamin A/C(N-18) (Cat#: sc-6215) was purchased from Santa Cruz (Dallas, Texas, USA). Mouse monoclonal anti-Nup93 (E-8) (Cat#: sc-374399) was purchased from Santa Cruz (Dallas, Texas, USA). Human anti-centromere CREST antibody(Cat#: 15–234) was purchased from Antibodies Incorporated (Davis, CA, USA). Rabbit-anti-p-Akt (s473) (Cat#: 193H12) was purchased from Cell Signaling Technology (Beverly, MA, USA). Rabbit anti-Akt (Cat#: SAB4500802) was purchased from Sigma (St. Louis, MO, USA). Rabbit anti-ZP3 (Cat#: AV35855) was purchased from Sigma (St. Louis, MO, USA). Rabbit anti-DIAPH2 (Cat#: orb213850) was purchased from biorbyt (San Francisco, CA, USA). Rabbit anti-PTPRK (Cat#: orb338638) was purchased from biorbyt (San Francisco, CA, USA). Rabbit anti-AIPL1 (Cat#: 15108-1-AP) was purchased from proteintech (Chicago, Illinois, USA). Cy2-conjugated donkey anti-mouse IgG (Code: 715-225-150), Cy2-conjugated donkey anti-rabbit IgG (Code: 711-225-152), fluorescein (FITC)-conjugated donkey anti-goat IgG (Code: 705-095-147), rhodamine (TRITC)-conjugated donkey anti-mouse IgG (Code: 715-025-150), rhodamine (TRITC)-conjugated donkey anti-goat IgG (Code: 715-025-147), rhodamine(TRITC)-conjugated donkey anti-human IgG (Code: 709-025-149) were purchased from Jackson ImmunoResearch Laboratory (WestGrove, PA, USA). Horseradish peroxidase (HRP)-conjugated goat anti-rabbit IgG and HRP-conjugated goat anti-mouse IgG were purchased from Vazyme (Nanjing, Jiangsu, China).

### Oocyte collection and culture

Immature oocytes arrested in prophase I (GV oocytes) were obtained from the ovaries of 3–4 week-old female ICR mice. The mice were sacrificed by cervical dislocation and ovaries were isolated and placed in operation medium (Hepes) with 2.5 nM milrinone and 10% fetal bovine serum (FBS) (Gibco). Oocytes were released from the ovary by puncturing the follicles with a hypodermic needle. Cumulus cells were washed off the cumulus-oocyte complexes and 50 isolated denuded oocytes were placed in 100-ul droplets of culture medium under mineral oil in plastic dishes (BD). Oocytes were cultured in MEM + medium (MEM with 0.01 mM EDTA, 0.23 mM Na-pyruvate, 0.2 mM penicillin/streptomycin, 3 mg/ml BSA and 20% FBS). Oocytes were cultured at 37.0 °C, in a 5% O_2_, 5% CO_2_ humidified atmosphere.

### siRNA production and transfection

DNA templates for siRNA and siRNA production are described in Supplementary File 1. For siRNA transfection, the N-TER^TM^ Nanoparticle siRNA Transfection System (Sigma) was used. Briefly, two tubes, one containing 1.1 μl N-TER^TM^ nanoparticles in 5.15 μl nuclease-free water (Acros Organics) and the other containing 1.625 μl of siRNA (5 μM) mixture in 4.625 μl of siRNA dilution buffer (provided by the kit) were gently mixed together and incubated at room temperature (RT) for 20 min. Next, the siRNA–nanoparticle complex solution was added to 100 μl of medium containing 50 oocytes. After 12–14 h, the oocytes were washed to remove the nanoparticle-containing medium. After 1–2 h, siRNA treatment was repeated, depending on how difficult the target was knocked down. 2.5 nM milrinone was added to cultures during siRNA treatment to prevent resumption of meiosis. Next, oocytes were transferred into milrinone-free MEM + and cultured for 8 or 16 h.

### Immunofluorescence

Oocytes were briefly washed in PBS with 0.05% polyvinylpyrrolidone (PVP), permeated in 0.5% Triton X-100/PHEM (60 mM PIPES, 25 mM Hepes pH 6.9, 10 mM EGTA, 8 mM MgSO_4_) for 5 min and washed three times rapidly in PBS/PVP. Oocytes were then fixed in 3.7% paraformaldehyde (PFA)/PHEM for 20 min, washed three times (10 min each) in PBS/PVP and blocked with blocking buffer (1% BSA/PHEM with 100 mM glycine) at RT for 1 h. Oocytes were then incubated overnight at 4 °C with primary antibody diluted in blocking buffer. After incubation, oocytes were washed three times (10 min each) in PBS with 0.05% tween-20 (PBST) and incubated at RT for 45 min with secondary antibody diluted in blocking buffer (1:750 in all cases). Cells were washed three times and DNA was stained by 10 μg/ml Hochest 33258 (Sigma). Oocytes were mounted onto a slide with mounting medium (0.5% propgal gallate, 0.1 M Tris-HCl, PH 7.4, 88% glycerol) and covered with a coverglass (0.13–0.17 μm thick). To maintain the oocyte dimensions tape was placed between the slide and cover glass. Primary antibody dilutions were as follows: anti-ZP3, 1:100; anti-tubulin, 1:500; anti-human centromere, 1:500; anti-Nup93, 1:200; anti-Lamin A/C, 1:100. Rhodamine-phalloidin (Cytoskeleton, Denver, CO, USA) was used at 1:200. The oocytes were examined with an Andor Revolution spinning disc confocal microscope (Oxford instruments, Belfast, Northern Ireland).

### Immunoprecipitation

For immunoprecipitation experiments, 5 μg control IgG or anti-ZP3 antibody was firstly coupled to 30 μl protein-A/G beads (Macgene) for 4 h at 4 °C on a rotating wheel in 250 μl IP buffer (20 mM Tris-HCl pH 8.0, 10 mM EDTA, 1 mM EGTA, 150 mM NaCl, 0.05% Triton X-100, 0.05% Nonidet P-40, 1 mM Phenylmethylsulfonyl fluoride) with 1:100 protease inhibitor (Sigma) and 1:500 phosphatase inhibitor (Sigma). Meanwhile, 600 ZP-free GV oocytes were lysised and ultra-sonicated in 250 IP buffer and then pre-cleaned with 30 μl protein-A/G beads for 4 h at 4 °C. Secondly protein A/G-coupled control IgG or anti-ZP3 antibody was incubated overnight at 4 °C with 250 μl pre-cleaned oocyte lysate supernatant. Finally the next morning beads were washed three times for 10 min each with 1 ml IP buffer and the resulting beads with bound immuno complexes were subjected to SDS-PAGE and silver staining.

### Silver staining and characterization of ZP3-interacting proteins

For silver staining, immuno-complexed beads from control IgG or anti-ZP3 antibody group were boiled in protein sample buffer and the supernatants were separated side by side on a SDS-PAGE gel and the gel were subsequently fixed overnight at 4 °C in fixing solution (10% acetic acid, 40% ethanol), sensitized 30 min at room temperature with fresh-made sensitizing solution (30% ethanol, 0.2% Na_2_S_2_O_3_, 0.314% Na_2_S_2_O_3_·5H_2_O and 6.8% sodium acetate) and washed three times with water for 5 min each. Then the gel was stained for 20 min at room temperature in fresh-made staining solution (0.25% AgNO_3_, 0.02% of fresh 37% formaldehyde solution), washed with water for 2.5 min and developed for about 5~10 min (depending on how fast the process is, avoid insufficient or excessive development) in developing solution (2.5% NaCO_3_, 0.02% of fresh 37% formaldehyde solution) and finally the developing reaction was stopped for 10 min in stopping solution (0.4% glycine).

For characterization of ZP3-interacting proteins, silver-stained control or ZP3 lanes were compared carefully and Those bands with significantly higher gray level in ZP3 lane were cut out one by one and store in protease-free tubes with 10% ethanol. Then the selected bands, which were potentially ZP3 interactors, were sent to Testing & Analysis Center, Nanjing Meidical University for MALDI-TOF-MS (Matrix-Assisted Laser Desorption/Ionization Time of Flight Mass Spectrometry). The identity of each protein was determined by PMF (Peptide mass fingerprinting) searching in Mascot (http://www.matrixscience.com/mascot/cgi/search_form.pl?FORMVER=2&SEARCH=PMF).

### Data analysis and statistics

All experiments were repeated at least three times, Measurement on confocal Images was done with Image J. Data were presented as x ±Sem. Statistical comparison between two groups was done with Student’s test of Excel. Statistical comparison between multiple groups was done with Duncan’s multiple range test of GraphPad. P < 0.05 was considered to be statistically significant.

## Discussion

In the current study, we hypothesized that ZP3 plays important roles in GVBD based on its localization patterns and the meiotic phenotype after ZP3 knockdown. Furthermore, we proposed a working model for ZP3-mediated GVBD based upon the identification of potential ZP3 interaction partners and thereby validated our model.

ZP3 exhibits a dynamic localization pattern and appears to interact with other ZP proteins. Structurally, although ZP3 has ZP domains similar to those of ZP1 and ZP2, regions beyond the ZP domains are quite different^26^,^27^. Newly synthesized cytoplasmic ZP3 appears to travel independently from ZP2, as the two proteins did not interact with each other before being targeted to the zona pellucida^28^,^29^. Investigators in a previous study localized ZP3 to the cell membrane in zona-free oocytes^30^, while in our study we observed ZP3 staining at the oolemma (oocyte membrane) and within the cytoplasm. We therefore hypothesize that ZP3 at the oolemma might be also be important since we observed significant changes in cortical F-actin organization after ZP3 knockdown. Interestingly, we also observed that ZP3 staining in the nucleus only partially overlapped with lamin A/C and Nup93. This indicated to us that ZP3 might exhibit other functions in addition to organization of nuclear lamins or nuclear pores.

We performed immunoprecipitation in lysates of ZP-free GV oocytes, which excluded the predominant interaction between ZP3 and other ZP proteins and enriched interactions between ZP3 and cytoplasmic proteins. We thereby identified Ptprk, Aipl1, and Diaph2 as interacting partners. Ptprk is a receptor-type protein tyrosine phosphatase that regulates beta-catenin in lung cancer cells^31^, and because Akt is upstream of beta-catenin in the cell cycle^32^, ZP3 might assist Ptprk in regulating Akt activity. Aipl1 is a photoreceptor-specific chaperone of the visual effector enzyme phosphodiesterase-6 (PDE6), and binds to and stabilizes the farnesylated PDE6A subunit^33^. Mutations in Aipl1 destabilized PDE6, which may be a cause of Leber congenital amaurosis type 4 (LCA4), a severe form of childhood blindness^34^. These findings suggest that ZP3 promotes Aipl1 binding and stabilization of farnesylated lamin to enhance lamin binding to the nuclear membrane, an events that precedes GVBD. Diaph2 contains two formin homolog domains that organize the actin cytoskeleton and a diaphanous GTPase-binding domain that binds to F-actin to inhibit actin polymerization^35^,^36^. Diaph2 also binds to microtubule end binding protein (EB1) and anaphase-promoting complex (APC) to stabilize microtubules^37^. Intriguingly, Diaph2 deficiency has been linked to premature ovarian failure in humans^38^. Based upon these findings, we hypothesize that ZP3 mediates Diaph2-regulated actin polymerization.

We have provided preliminary experimental evidence to validate our current model. We found that ZP3 knockdown greatly displaced lamin A/C from the nuclear envelop into the nucleus, suggesting that lamin A/C was not correctly bound to the nuclear envelope. Abnormal farnesylation of pre-lamin A was shown to disrupt localization to the nuclear membrane^8^,^9^. In human U2OS cells, constitutive farnesylation of lamin A, which is linked to progeria, inhibited the phosphorylation of Ser22 that is essential for lamin disassembly^8^. More importantly, ZP3 knockdown greatly diminished the nuclear concentrations of Aipl1, which is known to regulate farnesylation. From the collective evidence, we provide good support toward a role for ZP3 in regulating lamin distribution.

ZP3 knockdown increased F-actin intensity and decreased overall F-actin area in the oocyte cortex, indicative of altered actin dynamics. Normal actin dynamics are important for GVBD in bovine or *Xenopus* oocytes^17^,^18^, and an important role for actin dynamics has been confirmed for nuclear envelope breakdown in somatic cells^39^. We also found that ZP3 knockdown greatly diminished the nuclear concentration of Diaph2, which is known to play important roles in actin and microtubule dynamics. In addition, ZP3 knockdown significantly reduced p-Akt levels, which is reported to promote GVBD in mouse oocytes^40^,^41^.

Collectively, our findings have demonstrated that ZP3 is enriched in the nucleus and affects GVBD by regulating key kinases (particularly Akt, Aipl1 and Diaph2) that are important for cytoskeletal dynamics and organization. Further studies are required to define the mechanisms underlying these processes.

## Additional Information

**How to cite this article**: Gao, L.-L. *et al*. ZP3 is Required for Germinal Vesicle Breakdown in Mouse Oocyte Meiosis. *Sci. Rep.* **7**, 41272; doi: 10.1038/srep41272 (2017).

**Publisher's note:** Springer Nature remains neutral with regard to jurisdictional claims in published maps and institutional affiliations.

## Supplementary Material

Supplementary Information
