# Supplementary material for: ZP3 is Required for Germinal Vesicle Breakdown in Mouse Oocyte Meiosis
Source: Sci Rep. 2017 Feb 1;7:41272. doi: 10.1038/srep41272 (PMC5286536; doi:10.1038/srep41272)
Supplement: Supplementary Information [file srep41272-s1.pdf]

ZP3 is Required for Germinal Vesicle Breakdown in Mouse Oocyte Meiosis

Lei-Lei Gao[1, 2], Chun-Xiang Zhou[1, 2], Xiao-Lan Zhang[1], Peng Liu[1], Zhen Jin[1], Gang-Yi Zhu[1], Yang Ma[1], Jing Li[1], Zhi-Xia Yang[1, 3] and Dong Zhang [1, 3]

Methods

SiRNA design and production

DNA template sequences for siRNA production are listed in Table 1. The sequence of control templates was a mock sequence that does not specifically bind to any mRNA from the mouse genome. DNA templates against four different coding for DNA sequence (CDS) regions of ZP3 siRNA were designed online using BLOCK-iT™ RNAi Designer (http://rnaidesigner.invitrogen.com/rnaiexpress/) with some modifications. Sequence specificity was verified through a blast homology search.

SiRNAs were produced using the T7 RiboMAXTM Express RNAi System (Promega) according to the manufacturer's instructions. Briefly, for each double-stranded siRNA against one of the four ZP3 CDS regions, two pairs of synthesized complementary single-stranded DNA oligonucleotides were first annealed to form two double-stranded DNA templates. Subsequently, two complementary single-stranded siRNAs were separately synthesized in accordance with these two templates and then annealed to form a final double-stranded siRNA. Next, the siRNA was purified by conventional phenol/chloroform/isoproponal precipitation, which was then aliquoted and stored at −80°C after a quality check on an agarose gel. A ready-to-use siRNA mixture was prepared by mixing siRNAs against four target regions together at an equal molar ratio to a final concentration of 5 μM.

Table 1. DNA oligonucleotides for siRNA production.

Target site	DNA templates
ZP3 CDS 133-153[1]	Oligo1:GGATCCTAATACGACTCACTATAGAGTGTCTGGAAGCTGAACTA[2]
	Oligo2:AATAGTTCAGCTTCCAGACACTCTATAGTGAGTCGTATTAGGATCC[2]
	Oligo3:GGATCCTAATACGACTCACTATATAGTTCAGCTTCCAGACACTC[2]
	Oligo4:AAGAGTGTCTGGAAGCTGAACTATATAGTGAGTCGTATTAGGATCC[2]
ZP3 CDS 387-407[1]	Oligo1: GGATCCTAATACGACTCACTATAGACTAACCGTGTGGAGGTACC[2]
	Oligo2:AAGGTACCTCCACACGGTTAGTCTATAGTGAGTCGTATTAGGATCC[2]
	Oligo3: GGATCCTAATACGACTCACTATAGGTACCTCCACACGGTTAGTC[2]
	Oligo4:AAGACTAACCGTGTGGAGGTACCTATAGTGAGTCGTATTAGGATCC[2]
ZP3 CDS 727-747[1]	Oligo1:GGATCCTAATACGACTCACTATAGATGGTCTATCTGAGAGCTTT[2]
	Oligo2:AAAAAGCTCTCAGATAGACCATCTATAGTGAGTCGTATTAGGATCC[2]
	Oligo3: GGATCCTAATACGACTCACTATAAAAGCTCTCAGATAGACCATC[2]
	Oligo4:AAGATGGTCTATCTGAGAGCTTTTATAGTGAGTCGTATTAGGATCC[2]
ZP3 CDS 940-960[1]	Oligo1: GGATCCTAATACGACTCACTATAGAGGGTGATGCTGACATCTGT[2]
	Oligo2:AAACAGATGTCAGCATCACCCTCTATAGTGAGTCGTATTAGGATCC[2]
	Oligo3: GGATCCTAATACGACTCACTATAACAGATGTCAGCATCACCCTC[2]
	Oligo4:AAGAGGGTGATGCTGACATCTGTTATAGTGAGTCGTATTAGGATCC[2]
Control[3]	Oligo1: GGATCCTAATACGACTCACTATACCTACGCCACCAATTTCGTTT[2]
	Oligo2:AAAAACGAAATTGGTGGCGTAGGTATAGTGAGTCGTATTAGGATCC[2]
	Oligo3: GGATCCTAATACGACTCACTATAAAACGAAATTGGTGGCGTAGG[2]
	Oligo4:AACCTACGCCACCAATTTCGTTTTATAGTGAGTCGTATTAGGATCC[2]

1 The numbers are the starting and ending position of the target sites in ZP3 CDS (NM_011776.1 in NCBI).

2 two pairs of DNA oligos are needed for for each double-stand siRNA. Oligo 2 is complementary with oligo 1 except an "AA" overhang at 5'; Oligo 3 is complementary with oligo 4 except an "AA" overhang at 5'. In each oligo, gene-specific sequences are underlined, other sequences are for recognition and binding by T7 RNA polymerase.

3 Control siRNA does not target to any mRNA sequence in mouse.

Supplementary Figure 1

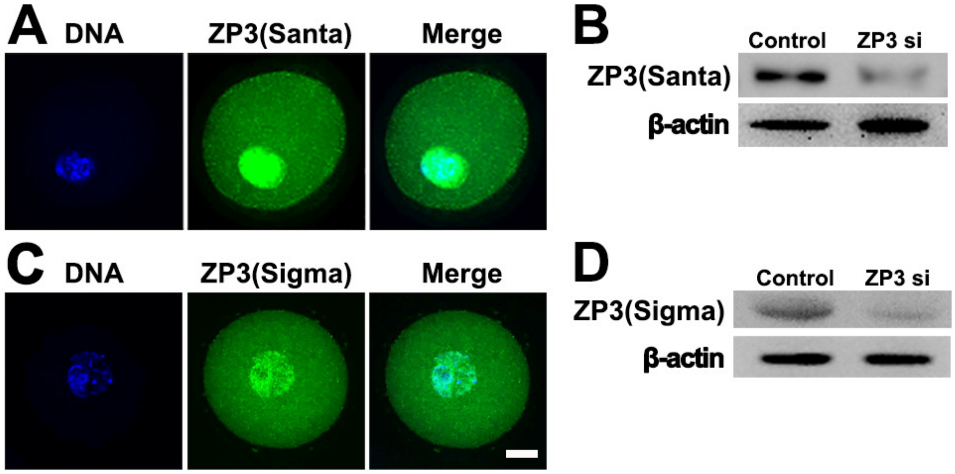

Supplementary Figure 2

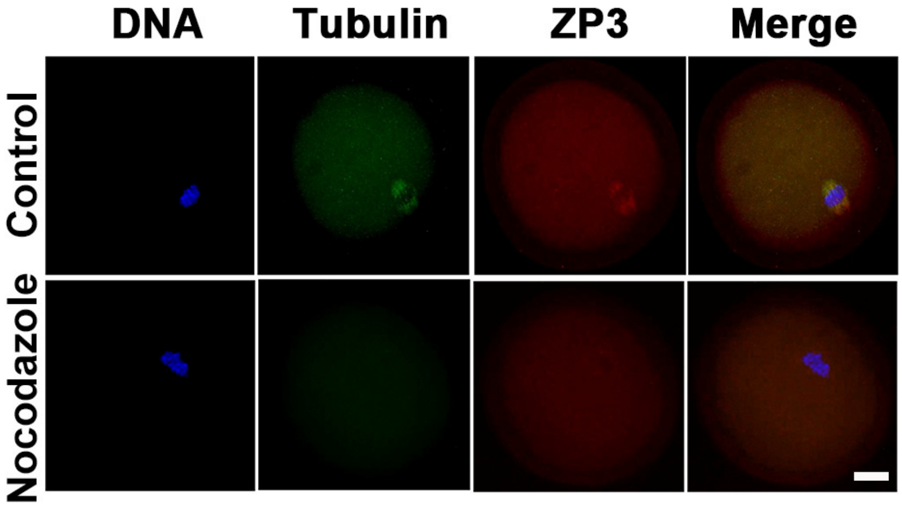

Figure S1. Anti-ZP3 antibodies from different companies had similar staining pattern.

A and B. Anti-ZP3 antibody from Santa Cruz detected strong nuclues stainig in GV oocytes. This ZP3 antibody is specific, as it detected a band at expected size and this band dramatically decreased after ZP3 is knocked downs by specific siRNA. **C and D.** Anti-ZP3 antibody from Sigma also detected strong nuclues stainig in GV oocytes. This ZP3 antibody is also specific, as it detected a band at expected size and this band also dramatically decreased after ZP3 is knocked downs by specific siRNA. DNA in blue, ZP3 in green. Scale bar, 20 μm.

Figure S2. The localization of ZP3 within spindle is microtubule-dependent.

At MI, ZP3 co-localized with spindle microtubules. After nocdazole treatment, microtubules largely disassembled and ZP3 localization within spindle also dramatically diminished. DNA in blue, tubulin in green, kinetchores in red. Scale bar, 20 μm.
